# Transcriptional Responses of the Heat Shock Protein 20 (Hsp20) and 40 (Hsp40) Genes to Temperature Stress and Alteration of Life Cycle Stages in the Harmful Alga *Scrippsiella trochoidea* (Dinophyceae)

**DOI:** 10.3390/biology9110408

**Published:** 2020-11-21

**Authors:** Yunyan Deng, Zhangxi Hu, Lixia Shang, Zhaoyang Chai, Ying Zhong Tang

**Affiliations:** 1 CAS Key Laboratory of Marine Ecology and Environmental Sciences, Institute of Oceanology, Chinese Academy of Sciences, Qingdao 266071, China; yunyandeng@qdio.ac.cn (Y.D.); zhu@qdio.ac.cn (Z.H.); lxshang@qdio.ac.cn (L.S.); zhaoyangchai@qdio.ac.cn (Z.C.); 2 Laboratory of Marine Ecology and Environmental Science, Qingdao National Laboratory for Marine Science and Technology, Qingdao 266237, China; 3 Center for Ocean Mega-Science, Chinese Academy of Sciences, Qingdao 266071, China

**Keywords:** *Scrippsiella trochoidea*, resting cyst, dinoflagellate, harmful algal blooms, heat shock protein 20 (Hsp20), heat shock protein 40 (Hsp40), temperature stress

## Abstract

**Simple Summary:**

As the greatest contributors to harmful algal blooms, dinoflagellates account for roughly 75% of bloom events, which become an escalating threat to coastal ecosystems and cause substantial economic loss worldwide. Resting cyst production and broad temperature tolerance are well proven as adaptive strategies for blooming dinoflagellates; however, to date, the underlying molecular information is scarce. In the present study, we characterized two heat shock protein genes from the representative dinoflagellate *Scrippsiella trochoidea*, with the aim to primarily determine their possible roles in response to temperature stress and alteration of the life cycle. The yielded results enhance our knowledge about the functions of cross-talk of different Hsp members in temperature adaptation of dinoflagellates and facilitate further exploration in their potential physiological relevance during different life-stage alternation in this ecological important lineage.

**Abstract:**

The small heat shock protein (sHsp) and Hsp40 are Hsp members that have not been intensively investigated but are functionally important in most organisms. In this study, the potential roles of a *Hsp20* (*StHsp20*) and a *Hsp40* (*StHsp40*) in dinoflagellates during adaptation to temperature fluctuation and alteration of different life stages were explored using the representative harmful algal blooms (HABs)-causative dinoflagellate species, *Scrippsiella trochoidea*. We isolated the full-length cDNAs of the two genes via rapid amplification of cDNA ends (RACE) and tracked their differential transcriptions via real-time qPCR. The results revealed *StHsp20* and *StHsp40* exhibited mRNA accumulation patterns that were highly similar in response to heat stress but completely different toward cold stress, which implies that the mechanisms underlying thermal and cold acclimation in dinoflagellates are regulated by different sets of genes. The *StHsp20* was probably related to the heat tolerance of the species, and *StHsp40* was closely involved in the adaptation to both higher and lower temperature fluctuations. Furthermore, significantly higher mRNA abundance of *StHsp40* was detected in newly formed resting cysts, which might be a response to intrinsic stress stemmed from encystment. This finding also implied *StHsp40* might be engaged in resting cyst formation of *S. trochoidea.* Our findings enriched the knowledge about possible cross-talk of different Hsp members in dinoflagellates and provided clues to further explore the molecular underpinnings underlying resting cyst production and broad temperature tolerance of this group of HABs contributors.

## 1. Introduction

Dinoflagellates are ubiquitous unicellular microalgae, which occupy vital niches in marine and freshwater environments [1]. Many members in this lineage have major ecological and economic impacts, among which the most notorious is resulting in harmful algal blooms (HABs) [1,2]. So far, approximately 200 species of dinoflagellates have been recorded to be able to cause HABs, accounting for roughly 75% of HAB events [3]. The ecological success of dinoflagellates under certain circumstances at least partly stems from their multiple ecological strategies and functional traits [4,5]. Some dinoflagellate species have a life-history strategy that includes a dormant stage in their life cycles, called resting cyst. Different lines of evidence prove that such cysts play an important role in the ecology of dinoflagellates, especially in those HABs-causative members, as they have been demonstrated to be associated with protecting cells from adverse environments, genetic recombination, seeding and terminating HABs, and geographic expansion of populations [5,6,7,8,9,10,11,12]. Additionally, some HABs-causative dinoflagellates are eurythermal species, which display wide tolerance to temperature fluctuations [13,14,15,16]. Several case studies have demonstrated that broad temperature tolerance contributed to population survival, distribution expansion, and even bloom formation of these species [15,16,17]. Despite the ecological importance of resting cyst production and broad temperature tolerance as ecological strategies for HABs-causative dinoflagellates, we currently have a poor understanding of their underlying genetic basis, such as the functional genes and the detailed molecular mechanisms involved, in large part owing to extremely larger and more complex genomes of dinoflagellates and relatively little attention paid to these facets.

Heat shock proteins (Hsps) are vital cellular chaperones, which function as central components in homeostasis maintenance under both favorable and unfavorable conditions and in coping with a variety of stresses [18,19]. The small Hsps (sHsps), which are a group of Hsps with molecular weights of 12–43 kDa, include an *α*-crystalline domain (ACD) and variable C- and N-terminal extensions [19,20]. Their monomers could aggregate into a large oligomer of approximately 200–800 kDa, which is essential for their function in aggregation prevention and new protein refolding [20,21]. The sHsp family probably contains the most diverse members in structure and function among various families of stress responsive proteins [22]. Their abundance and heterogeneity imply their unique and multiple functions [23]. Apart from stress resistance [24,25], sHsps have been proven to engage in a variety of physiological processes, including embryogenesis and cell proliferation [26,27,28], insect development [29], actin and intermediate filament dynamics [30,31], life span [32], membrane fluidity [33], and disease prevention [34,35,36]. The Hsp40s, characteristic with the presence of a J-domain, were initially identified to be capable of stimulating the ATPase activity of DnaK (the Hsp70 homolog in bacteria) and helping replicate phage DNA in host cells [37,38]. Until now, members in the Hsp40 family have been verified to play critical roles in a series of cellular protein processing, such as translation, folding, unfolding, translocation, and degradation [39,40,41,42]. As an important co-chaperonin of Hsp70s, Hsp40 recognizes unfolded substrates and delivers them to Hsp70, which in turn, leads to a conformational change in Hsp70 that stabilizes its binding to the substrates [39,43]. Many environmental cues, such as temperature and drought, are stimuli for Hsp40s production, and therefore, they are considered to be common cellular stress sensors and relevant to stress resistance [42,44,45]. Meanwhile, members of Hsp40s are also implicated in many aspects of Hsp70s functions, such as signal transduction, fungal growth, plant development, and various human diseases [39,42,46,47,48].

In dinoflagellates, Hsp-related investigations have progressed primarily from those on Hsp70 and Hsp90 families. Information about sHsps and Hsp40s is relatively scanty and fragmentary, as it mainly comes from omics-based resources without further structural and functional verification. Differential transcription levels were documented for *Hsp20* and *Hsp40* members in a *Symbiodinium* species subjected to thermal stress [49,50]. One *Hsp20* gene was once detected from *Karenia mikimotoi* and proposed to be involved in cell proliferation [51]. One DnaJ (Hsp40) chaperone homolog in *Prorocentrum donghaiense* was observed to be greatly upregulated upon dissolved inorganic phosphorus limitation [52]. DnaJ homologs were also recorded to display higher protein abundance in toxin-producing strains of dinoflagellate *Alexandrium catenella* than those in its non-toxic mutant, suggesting these homologs were involved in an adaptive response to active toxin biosynthesis in *A. catenella* [53]. Therefore, whether or not and how sHsp and Hsp40 in dinoflagellates are responding to temperature stress, as their names imply, remains to be important questions to be answered.

The dinoflagellate *Scrippsiella trochoidea* is an eurythermal (with a temperature tolerance range of 10–30 °C), cosmopolitan, HAB-forming, and toxic dinoflagellate [13,54,55]. Due to readily producing resting cysts, this species has been adopted as representative species for life history studies on dinoflagellates [56,57,58,59,60]. In the present work, the potential roles of a *Hsp20* (*StHsp20*) and a *Hsp40* (*StHsp40*) from *S. trochoidea* during adaptation to temperature fluctuation and alteration of different life stages were preliminarily investigated based on full-length cDNA isolation. The yielded results will enhance our knowledge about functions of Hsp members in dinoflagellates and facilitate further exploration of their possible ecological relevance in this lineage.

## 2. Materials and Methods

### 2.1. Scrippsiella trochoidea Culture Maintenance

The strain IOCAS-St-1 of *Scrippsiella trochoidea* originally isolated from the Yellow Sea of China was obtained from the Marine Biological Culture Collection Centre, Institute of Oceanology, Chinese Academy of Sciences and confirmed by rDNA sequencing [57]. The f/2 medium without silica [61] added with PII metal mix of GSe medium [62] and a penicillin–streptomycin solution (100×, Solarbio, Beijing, China) (final concentration of 2%) was made with autoclaved and sterile 0.22 μm filtered natural seawater with a salinity of 32–33. Cells used for culture maintenance were incubated at 20 ± 1 °C under 100 μmol photons m^−2^ s^−1^ provided with a light: dark cycle of 12: 12 h, using cool white fluorescent light.

### 2.2. Full-Length cDNAs Cloning of StHsp20 and StHsp40

For cDNA cloning, about 10^6^ fresh vegetative cells at the exponential growth stage from the regular cultures were harvested by centrifugation and used for total RNA extraction. Total RNA was isolated according to Deng et al. (2019) [58] and digested with RNase-Free DNase Set (QIAGEN, Hilden, Germany) following manufacture instruction. The concentration and quality of total RNA was determined by agarose gel electrophoresis and NanoDrop^TM^ 1000 spectrophotometer (Thermo Fisher Scientific, Waltham, MA, USA).

For fragments amplifications, single-strand cDNAs synthesized from ~1 μg total RNA with random primers using Reverse Transcriptase M-MLV (Takara, Tokyo, Japan) were used as templates. Two pairs of specific primers, 20-F and 20-R and 40-F and 40-R (Table 1), were designed based on *Hsp20*-like and *Hsp40*-like sequences, respectively, found in previous transcriptome of *S. trochoidea* (GenBank accession no. SRP058465; [57]) for amplifying cDNA fragments following the protocol described before [63]. To isolate the full-length cDNAs of the two genes, rapid amplification of cDNA ends (RACE) PCR were performed as described previously [58,63]. The obtained cDNA sequences from fragments amplifications were used to design gene-specific primers (Table 1) for 5′ and 3′ RACE, respectively. The first strand cDNAs prepared from ~1 μg total RNA with the anchor primer [58,63] were used as the templates in RACEs. In the 5′ RACEs, PCR was performed with forward primer DinoSL (specific to dinoflagellates; [64,65]) coupled with reverse primers 5r-20-outer and 5r-20-inner and 5r-40-outer and 5r-40-inner (Table 1). For the 3′ ends, PCR were run with the forward primers, 3r-20-outer and 3r-20-inner and 3r-40-outer and 3r-40-inner (Table 1), paired with reverse primer GeneRacer3 (Invitrogen, Karlsruhe, Germany). All final products were confirmed by agarose gel electrophoresis, purified, respectively, with Generay DNA fragment recovery kit (Shanghai, China), cloned into T-vector as reported [59] and then sequenced at the Sangon Biotech Company (Qingdao, China).

### 2.3. Sequence Analysis of StHsp20 and StHsp40

The generated full-length nucleotide sequences of *StHsp20* and *StHsp40* were analyzed with the ORF Finder program [66] and NCBI BLAST programs [67]. The molecular weights and theoretical isoelectric points were predicted using ProtParam software [68]. The secondary structures were identified with the SOPMA program [69]. The signal peptides and transmembrane topological structures were checked with SignalP 5.0 Server and TMHMM Server programs, respectively [70,71].

### 2.4. Samples Collection

#### 2.4.1. Temperature Stresses Exposure Treatments

To observe the transcriptional response of *StHsp20* and *StHsp40* exposed to temperature fluctuations, vegetative cells (approximately 1.5 × 10^4^ cells mL^−1^) grown at routine maintenance conditions (20 ± 1 °C) were aliquoted into culture plates (6-well; Corning, Corning, NY, USA). Temperature treatments of 3 scenarios were performed as described before [58,60] and briefed as follows: in the first assay, cells that were maintained at 20 °C were withdrawn and immediately treated with higher or lower temperature (30, 25, 20, 15, and 5 °C) for 60 min in incubators with the temperature set in advance; in the second time-course assay, cells that were maintained at 20 °C were exposed to 10 and 30 °C, respectively, for 0 (control; sampled at the start of exposure experiment), 3, 5, 10, 15, 20, 30, 60, 120, and 180 min in an incubator with pre-set temperatures; the last assay was conducted to compare the effects between a moderate and a drastic temperature stress. The drastic one was to expose the cultures directly to a temperature stress of 10 °C (i.e., from 20 to 30 °C and from 20 to 10 °C, respectively) for 60 min. While the moderate one was to expose the culture to a temperature stress of 5 °C for 10 min first and then expose to a further 5 °C change for 60 min (i.e., from 20 to 15 °C or 25 °C for 10 min and then to 10 or 30 °C for 60 min). For the abovementioned 3 assays, cultures kept at 20 °C were treated as control, and all treatments were conducted independently in triplicate. After treatments, approximately 2 × 10^4^ vegetative cells or resting cysts for each sample were harvested, immediately frozen in liquid nitrogen, and then kept at −80 °C.

#### 2.4.2. Cells at Different Stages of Life Cycle 

To investigate transcriptional response of *StHsp20* and *StHsp40* at different stages of the life cycle, vegetative cells and resting cysts were prepared, referring to the previous studies [58,59,60], and briefed below. For vegetative cells, *S. trochoidea* (initial cell density of ~2 × 10^3^ cells mL^−1^) was inoculated into 300 mL medium in 500 mL Pyrex flask. Cell density was determined via daily cell counting as described in Deng et al. (2019) [59]. The growth stages of vegetative cells were determined according to growth curve (see Supporting Information Figure S2 for more details). Vegetative cells (~2 × 10^4^ cells for each sample; 3 biological replicates) were harvested on day 5, 9 (exponential growth stage) and day 12, 15 (stationary growth stage) (the day of inoculation was recorded as day 0) for qPCR gene analysis.

For resting cyst samples, including newly formed resting cysts, resting cysts maintained at 4 °C in darkness for 20 and 30 days, respectively, were produced according to Deng et al. (2019) [58] and briefed as follows. Vegetative cells grown in 6-well culture plates (Corning, Corning, NY, USA; 10 mL in each well) were used for resting cyst production. They were incubated at routine maintenance conditions except for that the medium was made with artificial seawater supplemented with all nutrients of the abovementioned recipe but nitrogen and phosphorus [58]. For the species, *Scrippsiella trochoidea*, there are clear distinctions in morphological features between vegetative cells and resting cysts (Supporting information Figure S1). Vegetative cells with their two flagella swam in the plates, while resting cysts without flagellum settled at the bottom, which allowed a swift judgement and separation via light microscopy (see [57] for more details). Cyst formation was checked every other day under inverted microscope (Olympus IX73). Resting cysts were generally obtained from the cultures that had been inoculated for about 20–30 days. They were cleaned with fresh sterile filtered seawater several times until no vegetative cell or planozygote (motile zygote) was observed under microscope. The obtained resting cysts (in 6-well culture plates) were then stored at 4 ± 1 °C in darkness for 20 and 30 days, respectively. For each sample, approximately 2 × 10^4^ vegetative cells or resting cysts (3 biological replicates) were harvested, immediately frozen in liquid nitrogen, and then kept at −80 °C.

### 2.5. Real-Time Quantitative PCR (qPCR) 

The qPCRs were performed on Bio-Rad CFX96 Real-Time PCR Detection System using Takara TB Green Premix Ex Taq™ II (Tokyo, Japan). The protocol and cycling conditions generally followed that described in [60]. Samples (~2 × 10^4^ cells or cysts for each sample; 3 biological replicates) prepared as described in 2.4 were used in qPCR detection. Total RNAs were extracted as mentioned above. For each sample, the first-strand cDNA synthesized from ~60 ng total RNA with random primers was used as template. The qPCR was performed in a volume of 20 μL, which contained 10.0 μL 2×SYBR Premix (TaKaRa, Tokyo, Japan), 1 μL template cDNA, 0.5 μL of each primer (10 μM), and 8.0 μL RNase-free water. The cycling conditions were 95 °C for 30 s, followed by 40 cycles of 94 °C for 5 s, 50 °C for 30 s, and 72 °C for 30 s. Two specific primer sets, q20-F and q20-R and q40-F and q40-R (Table 1), were used to amplify 183 bp and 124 bp products of *StHsp40* and *StHsp20*, respectively. The combination of *UBQ* (ubiquitin), *UBC* (ubiquitin conjugating enzyme), and *MDH* (malate dehydrogenase) was used as internal control in the qPCR detection of cells exposure to temperature stresses [60]. The combination of *MDH*, *UBC*, and *LBP* (luciferin-binding protein) was applied into qPCR analyses of genes transcription at different stages of the life cycle [57]. All qPCR products were sequenced to confirm correct amplifications. For each primer set, relative standard curve [72] and qPCR efficiency (*E*) [73] were determined as previously described [58]. Relative quantitative values were calculated by the 2^−△△Ct^ relative quantification method with qBasePlus software [74,75]. All reactions were performed in biological triplicates. The relative expression levels of targeted genes among different treatments were analyzed using one-way analysis of variance (ANOVA), with the significant level of *P* set at 0.05. All statistical tests were performed with SPSS 20.0 statistical software.

## 3. Results

### 3.1. cDNA Cloning and Sequencing Characterization of StHsp20 and StHsp40

**Figure 1:**
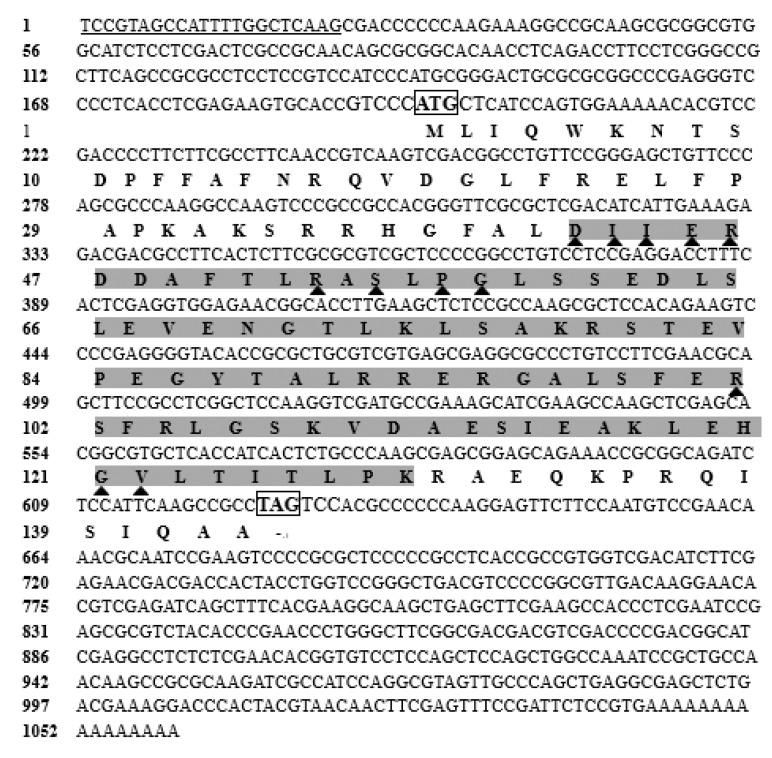
The cDNA and deduced amino acid sequences of *StHsp20* (accession number: MN698834): the boxes for start and stop codons; underline for DinoSL; dark gray background for characteristic α-crystalline domain (ACD); triangles for the putative dimer interfaces.

The full-length cDNA of *StHsp20* was 1059 bp, comprising a 194 bp 5′-UTR with the canonical dinoflagellate spliced leader (DinoSL) sequence, a 433 bp 3′-UTR, and a 432 bp ORF (open reading frame) (GenBank accession no. MN698834). The *StHsp20* ORF had 63% G + C content and encoded 143 amino acids, with a predicted molecular weight of 16.09 kDa and an isoelectric point of 9.39. The results showed that the deduced amino acid sequence containing a characteristic *α*-crystalline domain (ACD) spanned 88 amino acid residues (42–192 aa) (Figure 1). Twelve amino acid residues, including Asp42, Ile43, Ile44, Glu45, Arg46, Arg53, Ser55, Pro57, Gly58, Arg101, Gly121, and Val122, were predicted to compose the putative dimer interface in the ACD (Figure 1). No signal peptide was detected via the SignalP program. Moreover, the *StHsp20* protein was predicted to present outside membrane and contain 25.17% *α*-helix, 18.88% extended strand, 8.39% *β*-turn, and 47.55% random coil.

**Figure 2:**
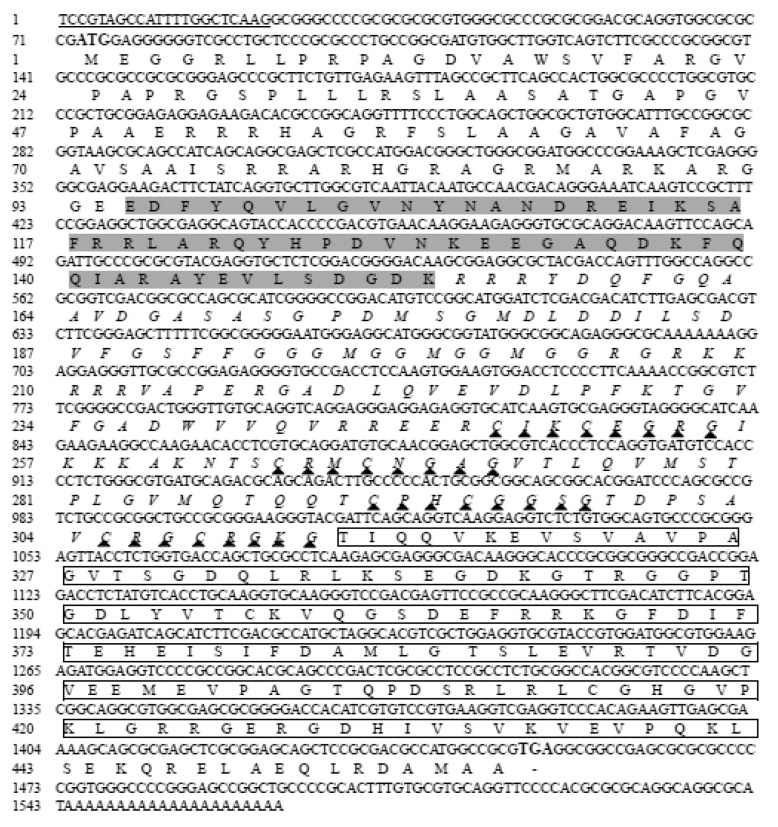
The cDNA and deduced amino acid sequences of *StHsp40* (accession number: MN698835): boldface for start and stop codons; underline for DinoSL; dark gray background for the characteristic J-domain (95–153 aa); triangles for the typical 4 repeats of CxxCxGxG in the predicted zinc-finger domain (154–312 aa; italics); box for the putative C-terminal domain (313–442 aa).

The assembled full-length *StHsp40* (1564 bp; GenBank accession no. MN698835) covered a 112 bp 3′ UTR and a 72 bp 5′ UTR with a conserved DinoSL sequence. The resultant 1380 bp ORF with 66% G + C content encoded a protein of 459 amino acids, with a predicted molecular weight of 49.22 kDa and an isoelectric point of 9.58. The BLAST results showed that the StHsp40 protein included 3 characteristic domains: a classical J-domain (95–153 aa), a zinc-finger domain (154–312 aa) featured by four repeats of CxxCxGxG, and a C-terminal domain (313–442 aa) (Figure 2). The SignalP program analysis found no signal peptide in StHsp40. According to the transmembrane topological structure analysis using TMHMM Server program, StHsp40 was presumed to be outside membrane. Four conformational states, 27.45% *α*-helix, 18.52% extended strand, 7.19% *β*-turn, and 46.84% random coil, were predicted in the secondary structure, suggesting that random coil is the major component of StHsp40.

### 3.2. Transcriptional Responses of StHsp20 and StHsp40 to Temperature Stresses

**Figure 3:**
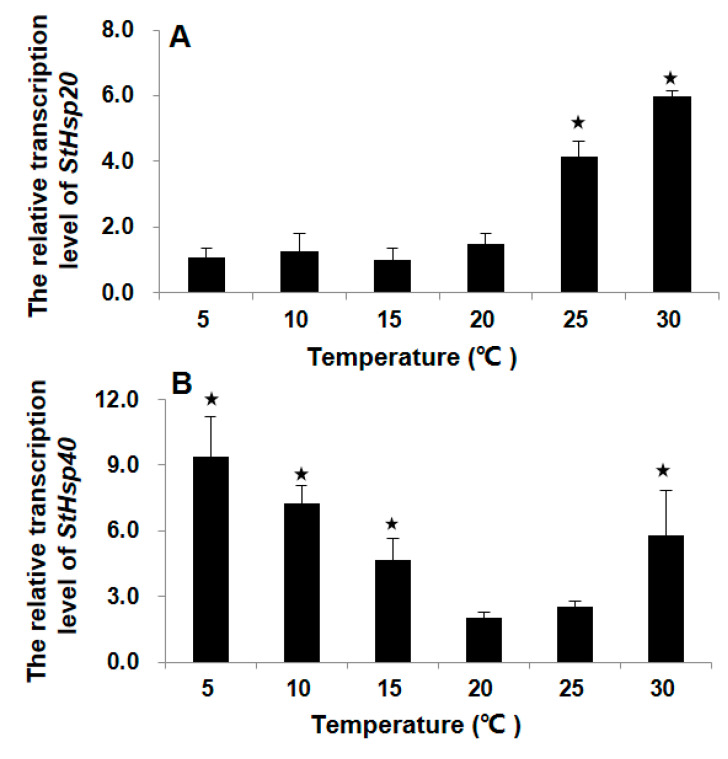
Transcription profiles of *StHsp20* (**A**) and *StHsp40* (**B**) in cells with different temperature treatments. The treatment groups that are significantly different (*p* < 0.05) relative to the control (20 °C group) are noted with asterisks. Error bars represent the standard deviation from 3 replicates.

For *StHsp20*, after thermal treatments (25 or 30 °C) for 1 h, markedly elevated transcripts were detected as compared to that in 20 °C control (ANOVA, *p* < 0.01; Figure 3A), while, however, no significant variation in transcripts was found in the treatments of lower temperatures (15, 10, or 5 °C) (ANOVA, *p* > 0.05; Figure 3A). For *StHsp40*, significant augments were detected in *StHsp40* transcripts after being subjected to both lower (5, 10, or 15 °C) and higher (30 °C) temperature stresses (ANOVA, *p* < 0.01; Figure 3B). The lowest transcription was observed at 20–25 °C, and the transcriptions elevated with the magnitude of temperature changes along both the decreasing and increasing directions (Figure 3B).

**Figure 4:**
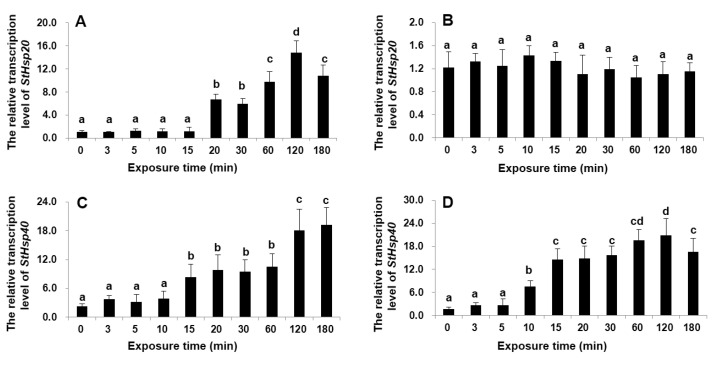
Transcription profiles of *StHsp20* (**A**,**B**) and *StHsp40* (**C**,**D**) in cells exposed to 30 °C (**A**,**C**) and 10 °C (**B**,**D**) with durations from 3 to 180 min. Different letters show significant differences (*p* < 0.05). Error bars represent the standard deviation from 3 replicates.

For the mRNA accumulations over time in the exposure to a change of ±10 °C, the mRNA expressions of both *StHsp20* and *StHsp20* in response to a 10 °C increase displayed a clearly time-dependent response with a similar trend during the 180 min exposure: the *StHsp20* expression was significantly upregulated from 20 min and on and reached the highest at 120 min (~14-fold), and then declined (ANOVA, *p* < 0.01; Figure 4A); the *StHsp40* expression elevated significantly from 15 to 180 min (peak), with the peak being ~9-fold higher than that measured at the beginning of experiment (ANOVA, *p* < 0.01; Figure 4C). In responses to a decrease of 10 °C, the *StHsp20* mRNA accumulation was statistically similar to that in the 20 °C control throughout the entire 180 min exposure (ANOVA, *p* > 0.05; Figure 4B), while the *StHsp40* expression was significantly upregulated from 10 min exposure and so on, with the peak observed at 120 min, which then decreased (ANOVA, *p* < 0.01; Figure 4D).

**Figure 5:**
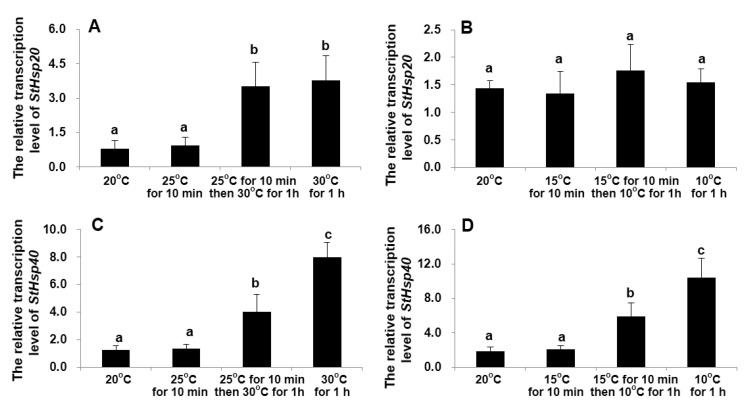
Transcription profiles of *StHsp20* (**A**,**B**) and *StHsp40* (**C**,**D**) in cultures exposed to one-step or stepwise temperature stress (+10 °C (**A**,**C**); −10 °C (**B**,**D**). Different letters show significant differences (*p* < 0.05). Error bars represent the standard deviation from 3 replicates.

In the experiment designed to compare the transcriptional responses between a moderate temperature stress (a 5 °C increase or decrease for each step) and a drastic temperature shock (a 10 °C increase or decrease in one step), the transcript levels of the two genes were not significantly stimulated by the moderate stress (± 5 °C) within the first 10 min (ANOVA, *p* > 0.05; Figure 5). Both the moderate and drastic stresses for 1 h greatly stimulated the mRNA accumulation of *StHsp20* by more than four-fold (ANOVA, *p* < 0.01; Figure 5A), with the transcription levels in the two groups statistically being the same (ANOVA, *p* > 0.05; Figure 5A). Neither stepwise nor one-step decreasing temperature shocks could markedly affect its transcription (ANOVA, *p* > 0.05; Figure 5B). For *StHsp40*, the transcriptional levels were significantly elevated in response to the drastic 10 °C shock, compared to the stepwise stresses (ANOVA, *p* < 0.01; Figure 5C,D).

### 3.3. Transcriptional Responses of StHsp20 and StHsp40 at Different Stages of Growth and Life Cycle

**Figure 6:**
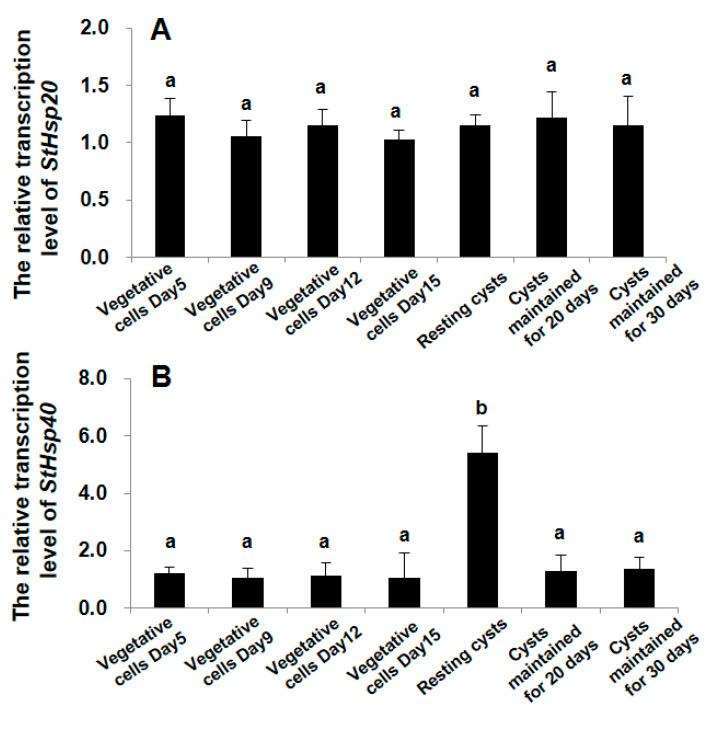
Transcription profiles of *StHsp20* (**A**) and *StHsp40* (**B**) in cells or resting cysts at different stage of life cycle. Different letters show significant differences (*p* < 0.05). Error bars represent the standard deviation from 3 replicates.

Our results clearly demonstrated different responses of the two genes in responding to the alteration of life cycle stages of *S. trochoidea*. The expression of *StHsp20* was observed to be statistically the same among all groups of the vegetative cells and resting cysts (ANOVA, *p* > 0.05; Figure 6A). On the striking contrast, the mRNA abundance of *StHsp40* in newly formed resting cysts was observed to be significantly higher than those in all other groups (ANOVA, *p* < 0.01; Figure 6B), and no significant difference in mRNA abundance was observed among all other groups (ANOVA, *p* > 0.05; Figure 6B). 

## 4. Discussion

### 4.1. Structural Characterization of StHsp20 and StHsp40

For the StHsp20 obtained from *S. trochoidea* in this study, the characteristic ACD is in accordance with the structural characteristics of other sHsp members [19,21,22,76]. The sHsp family probably contain the most diverse members in structure and function among various families of stress-responsive proteins [22]. Although their amino acid sequences are not as well conserved as those of the high molecular mass Hsps, plant sHSP monomers are all encoded by nuclear genes and share a conserved ACD located in the C- terminal [19,20]. Their N-terminals are highly variable, which is important both in chaperone activity and substrate specificity [76]. The founding members that were given the name Hsp40s contain three characteristic domains: the J-domain, which is the typical domain for the Hsp40 family and responsible for the interaction with Hsp70; the zinc-finger domain, which is different from other canonical zinc-finger domains, characterized by four repeats of CxxCxGxG, and is associated with protein-protein interactions; the C-terminal domain, which facilitates dimerization and also participates in interactions with the substrates [39,41]. According to the presence or absence of these three domains, Hsp40 proteins have been classified into three groups: DNAJA group (retain all three domains like the original DnaJ protein of *E. coli*), DNAJB group (lack the zinc-finger domain), and DNAJC group (cover the J-domain only) [38,41,43]. The StHsp40 obtained from the dinoflagellate *S. trochoidea* in the present work contains all three structural domains as initially described in *E. coli* DnaJ, indicating our StHsp40 belongs to the DNAJA group. The conserved domains found in StHsp20 and StHsp40 imply that the functions of Hsp20 and Hsp40 may possibly be conserved among organisms of different taxa, and/or StHsp20 and StHsp40 function in the *S. trochodea* (and other dinoflagellates) the same way as identified from organisms of other evolutionarily distant groups.

### 4.2. Transcriptions of StHsp20 and StHsp40 in S. trochoidea Cells in Responding to Temperature Shocks

The Hsps have been known to facilitate environment adaptation of organisms to various unfavorable scenarios potentially causing cellular damage by preventing cells from formation and accumulation of abnormal proteins within cells [18,77]. Temperature fluctuation or shock is a typical stimulus for Hsps production [78]. In the present study, the *StHsp20* from *S. trochoidea* exhibited transcription profiles that were completely different between thermal (“heating”) and cold (“cooling”) stresses: the shocks of higher temperatures markedly increased the transcripts of *StHsp20* but lower temperatures (5–15 °C) did not, suggesting *StHsp20* may indeed function as a “heat shock protein” in the subsequent cellular defense to heat stress, as implied in the gene name. This is consistent with the observations in previous investigations on *Arabidopsis* [20], diatom [79], oyster [80], copepod [81], rotifer [82], and insect [83]. In the time-course tests (i.e., exposure to 30 or 10 °C for 0–180 min), the 10 °C increment shock elicited drastically higher mRNA levels of *StHsp20* after 20 min exposure and maintained levels higher than those in the initial period from 0 to 15 min. Our recent work also observed rapid and significant increases in the mRNA amounts of *Hsp60* and *Hsp10* in *S. trochoidea* within 5 and 10 min, respectively, after exposing cells to temperature shocks from 20 to 30 °C and from 20 to 10 °C [58], implying that *Hsp20*, *Hsp60*, and *Hsp10* are all involved in thermal adaptation of the species but a more urgent or sensitive response in *Hsp60* and *Hsp10*. These results together also imply that the temperature adaptation of dinoflagellates is accurately controlled by an orchestrated transcription of different Hsps. Lower-temperature stress (a decrease from 20 to 15, 10 or 5 °C), regardless of a moderate or drastic stress, however, did not significantly stimulate the transcription of *StHsp20*, which is different from the responses of *Hsp60* and *Hsp10* [58], indicating *StHsp20* not a gene involved in cold shock adaptation. These results may also imply that the thermal and cold adaptation/acclimation in dinoflagellates are controlled by mechanisms and components that are associated by different. The *StHsp20* was probably related to the heat tolerance of the species. 

The transcript levels of *StHsp40* were greatly stimulated by both thermal and cold inducement within a time period of at least 3 h, regardless of moderate change or drastic shocks. This result suggested that the *StHsp40* probably plays roles in stabilizing and protecting proteins during both heat and cold stresses in *S. trochoidea*, akin to previously confirmed temperature responses of *Hsp40*s in other organisms by multiple investigations [42,44,45]. We detected significantly elevated *StHsp40* transcriptions in cells exposing higher (+10 °C) and lower (−10 °C) temperatures after 15 and 10 min, respectively. Comparing results between the moderate (5 °C change for each step) and drastic (10 °C change for one step) temperature stress, *StHsp40* transcripts were observed to be significantly higher in the latter groups. Our previous work on *Hsp70* in another HAB-forming dinoflagellate *Akashiwo sanguinea* found that the same drastic temperature shocks significantly stimulated its transcription within 10 min, and it was also higher than that observed in the moderate changes in temperature [63]. These results demonstrated that *Hsp40* and *Hsp70* in dinoflagellates have similar transcriptional responses to temperature stresses. It was previously inferred that *Hsp40* may functionally interact with *Hsp70* in the same heat shock pathway in dinoflagellates, where Hsp40 primarily acts as coupling factors to stimulate ATP hydrolysis of their chaperone proteins, Hsp70s [39,40,43]. Our results together support this inference and suggest *StHsp40* is closely involved in the adaptation to both higher and lower temperature fluctuations of *S. trochoidea*.

### 4.3. Differential Transcriptional Responses of StHsp20 and StHsp40 to the Alteration of Life Cycle Stages (Vegetative Cells vs. Resting Cysts)

Although Hsp proteins were initially known for heat shock response, evidence available in the literatures has indicated that various stressors other than temperature could stimulate their transcriptions, including, indeed, almost all known stresses [77,78]. For dinoflagellates, together with some other free-living protists, the formation of cysts is an adaptive strategy to survive in stressful conditions [5,57,84]. This study, however, did not observe significant transcriptions of *StHsp20* changing with alterations of growth stages of vegetative cells and that of life cycle stages (from vegetative cells to resting cysts), suggesting the new generated *StHsp20* might not function during resting stage formation and persistence of the species. Many higher plants have more than 20 sHsps, forming a more diverse family than other Hsps with respect to sequence similarity, cellular location, and functions [18]. The presence of abundant and heterogenous sHSPs suggests that they may have different or various physiological functions [23]. Therefore, further characterization of more sHsps from more dinoflagellate species at different life cycle stages is highly desirable for a more insightful understanding about their biological and ecological functions in dinoflagellates.

Apart from functioning in protecting cells from deleterious stresses, Hsps are proven to constitutively express at normal growth temperatures and play essential and indispensable roles in the life cycle [77,78]. Their transcriptions can be evoked even by non-environmental cues such as genetic stress [18,77,85]. Resting cyst of dinoflagellate is a benthic, dormant stage usually resulting from sexual reproduction. Sexual reproduction, which may increase genetic variance affect the ecology and evolution of species, is thus a kind of potential genetic stress [77,85]. In the present study, drastically elevated *StHsp40* transcription was detected in newly formed resting cysts, compared to those in vegetative cells and those in resting cysts maintained in the cold for a longer time, which might be viewed as a response to intrinsic stress resulting from sexual reproduction and encystment. It is also suggested that *StHsp40* might have functions in the resting cyst formation of *S. trochoidea*. This transcription pattern is also consistent with observations in mouse, yeast, fruit fly, *Haliotis asinine*, and mammalian cells, in the sense that Hsps transcriptions were activated upon exposure to genetic stresses such as inbreeding [85,86], senescence [87], deleterious mutations [88,89], and morphogenesis [77,90,91].

In nature, many species are exposed to hostile environmental conditions, such as high temperatures and high levels of toxicity. It is a common phenomenon that individuals at certain life stage are prone to being more vulnerable to stressful environments, a typical example is animals at the juvenile stage wherein mobility is often low and behavioral avoidance is limited. Studies on several organisms have found that occupation of different environments for different life stages might select for life-stage-specific Hsp expression and resistance [77]. Works on seeds of higher plants revealed that Hsp members, such Hsp70 and sHsp, were upregulated at transcriptional level by dormant stage-inducing conditions in the absence of abiotic stress [92]. Their protein abundance was also recorded to be higher in dormant stage compared to that in non-dormant stage and significantly decreased during germination [93]. The prominent expression of these genes and proteins were proposed to be part of the seed developmental program, which are required to ensure the proper folding of other proteins at the dormant state [92,93]. A paralleled finding was observed in the dinoflagellate *Akashiwo sanguinea*, of which the *Hsp70* transcription peaked in newly formed resting cysts undergoing morphogenesis and then dropped to lower levels in more mature (i.e., long-stored) cysts [63]. Our recent work on *S. trochoidea* also detected significantly higher transcriptions of *Hsp60* and *Hsp10* in resting cysts (both newly formed and long-stored cysts) relative to that in vegetative cells [58]. All of these results seem to imply some relationship between Hsps and life history traits of dinoflagellates; however, detailed knowledge is still in its infancy. It can be the starting point for future research to understand the physiological relevance of Hsps in this unique group of eukaryotes. Several gene expression studies have shown that dinoflagellates may rely heavily on posttranscriptional mechanisms [94,95]. Therefore, future works on more Hsp members in dinoflagellates under various stresses and developmental procession from both the transcriptional and translational levels may help us to enrich the knowledge about their ecological implications in relation not only to environmental cues in general but also to genetic stress of intrinsic nature in this unique lineage of eukaryotes.

## 5. Conclusions

In summary, we used the cosmopolitan, toxic, and resting-cyst-producing dinoflagellate, *Scrippsiella trochoidea*, as a representative species of HAB-forming dinoflagellates to probe the potential roles of *Hsp20* and *Hsp40* genes in dinoflagellates during adaptation to temperature fluctuation and alteration of different life stages. We found that *StHsp20* and *StHsp40* exhibited highly similar mRNA accumulation patterns when exposed to heat stresses but completely different toward cold stresses, implying the mechanisms underlying thermal and cold acclimation in dinoflagellates are regulated by different sets of genes. The *StHsp20* was probably related to the heat tolerance of the species and *StHsp40* was closely involved in the adaptation to both higher and lower temperature fluctuations. Furthermore, we found *StHsp40* displayed a significantly higher transcriptional level in newly formed resting cysts, which might be a response to intrinsic stress stemmed from encystment and also implied *StHsp40* might be directly involved in resting cyst formation of *S. trochoidea*. Although the data we acquired here are still a long way from being speculatedthe possible application into prevention, control, and mitigation of HABs, our findings provide clues to understand the molecular underpinnings underlying the two well-documented adaptive strategies of HABs-forming dinoflagellates, which might provide theoretical support for improving HABs management strategies and response in the future.

## Figures and Tables

**Table 1:** Primers details.

Primer Name	Nucleotide Sequences (5′→3′)	Amplicon Length
40-F	GGCGAGGAAGACTTCTATCAGG	587 bp
40-R	TGCTGCGTCTGCATCACG	
5r-40-outer	CAATCTGCTGGAACTTGTCCTGCG	496 bp
5r-40-inner	GCGGACTTGATTTCCCTGTCGTTG	416 bp
3r-40-outer	AGTGGACCTCCCCTTCAAAACCG	830 bp
3r-40-inner	CCACCCCTCTGGGCGTGATGC	670 bp
20-F	CCCTTCTTCGCCTTCAACC	216 bp
20-R	TTCTGTGGAGCGCTTGG	
5r-20-outer	GCTTCAAGGTGCCGTTCTCCA	417 bp
5r-20-inner	CGTGGCGGCGGGACTTGG	306 bp
3r-20-outer	CGACATCATTGAAAGAGACGACGCC	750 bp
3r-20-inner	TTCACTCGAGGTGGAGAACGGCA	680 bp
q40-F	GCATCAAGTGCGAGGGTAGG	124 bp
q40-R	TGCTGCGTCTGCATCACG	
q20-F	TGGAGAACGGCACCTTGAA	183 bp
q20-R	TGGGCAGAGTGATGGTGAGC	

## Supplementary Materials

The following are available online at https://www.mdpi.com/2079-7737/9/11/408/s1, Figure S1: Morphological observations for (A) vegetative cell and (B) resting cyst of *Scrippsiella trochoidea* under light microscope, Figure S2: Growth curve of vegetative cells of *Scrippsiella trochoidea*.
