# Supplementary material for: Transcriptional Responses of the Heat Shock Protein 20 (Hsp20) and 40 (Hsp40) Genes to Temperature Stress and Alteration of Life Cycle Stages in the Harmful Alga Scrippsiella trochoidea (Dinophyceae)
Source: Biology (Basel). 2020 Nov 21;9(11):408. doi: 10.3390/biology9110408 (PMC7700488; doi:10.3390/biology9110408)
Supplement: Supplementary file 1 [file biology-09-00408-s001.pdf]

Supplementary materials: Transcriptional Responses of the Heat Shock Protein 20 (Hsp20) and 40 (Hsp40) Genes to Temperature Stress and Alteration of Life Cycle Stages in the Harmful Alga *Scrippsiella trochoidea* (Dinophyceae)

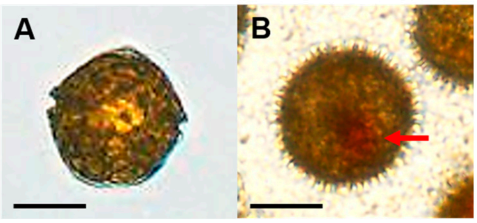

Figure S1. Morphological observations for (**A**) vegetative cell and (**B**) resting cyst of *Scrippsiella trochoidea* under light microscope. Scale bar = 20 μm. A. Vegetative cell usually conical epitheca, round hypotheca, and apical process; B. resting cyst usually round or oval shaped with red body (arrow) and numerous surface spines.

To prepared vegetative cells at different growth stages, *Scrippsiella trochoidea* (initial cell density of ~ 2×10^3 cells mL^{-1}) was inoculated into 300 mL medium in 500 mL Pyrex flask and were grown under routine culturing conditions (n = 3). The experiments lasted 17 days, 0.5 mL of culture sample (3 biological replicates) was taken every day and fixed with Lugol's solution (3% final concentration) for enumeration under an inverted microscope using a 1 mL Sedgewick Rafter counting chambers. The growth curve (Figure S2) was determined via daily cell counting (the day of inoculation was recorded as day 0). Vegetative cells were harvested on Day 5, 9 (exponential growth stage) and Day 12, 15 (stationary growth stage) for qPCR gene analysis. For each sample, ~ 2×10^4 cells with 3 biological replicates were prepared.

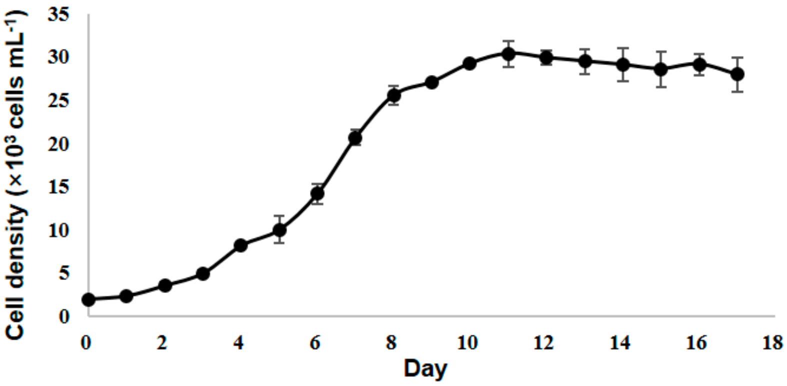

Figure S2. Growth curve of vegetative cells of *Scrippsiella trochoidea* strain IOCAS-St-1. Values are mean ± standard deviation, *Error Bars* = SD, n = 3.
